# Effects of *Prunus cerasus* L. Seeds and Juice on Liver Steatosis in an Animal Model of Diet-Induced Obesity

**DOI:** 10.3390/nu12051308

**Published:** 2020-05-04

**Authors:** Ilenia Martinelli, Maria Vittoria Micioni Di Bonaventura, Michele Moruzzi, Consuelo Amantini, Federica Maggi, Maria Gabriella Gabrielli, Alessandro Fruganti, Andrea Marchegiani, Fabrizio Dini, Carlotta Marini, Carlo Polidori, Giulio Lupidi, Francesco Amenta, Seyed Khosrow Tayebati, Carlo Cifani, Daniele Tomassoni

**Affiliations:** 1 School of Pharmacy, University of Camerino, 62032 Camerino, Italy; ilenia.martinelli@unicam.it (I.M.); mariavittoria.micioni@unicam.it (M.V.M.D.B.); carlo.polidori@unicam.it (C.P.); giulio.lupidi@unicam.it (G.L.); francesco.amenta@unicam.it (F.A.); khosrow.tayebati@unicam.it (S.K.T.); 2 Department of Medicine, University of Leipzig, 04103 Leipzig, Germany; michele.moruzzi@medizin.uni-leipzig.de; 3 School of Biosciences and Veterinary Medicine, University of Camerino, 62032 Camerino, Italy; consuelo.amantini@unicam.it (C.A.); gabriella.gabrielli@unicam.it (M.G.G.); alessandro.fruganti@unicam.it (A.F.); andrea.marchegiani@unicam.it (A.M.); fabrizio.dini@unicam.it (F.D.); carlotta.marini@unicam.it (C.M.); 4 Department of Molecular Medicine, University “Sapienza”, 00185 Rome, Italy; federica.maggi@uniroma1.it

**Keywords:** diet-induced obese rats, liver, obesity, tart cherry, anthocyanins

## Abstract

The accumulation of adipose tissue increases the risk of several diseases. The fruits-intake, containing phytochemicals, is inversely correlated with their development. This study evaluated the effects of anthocyanin-rich tart cherries in diet-induced obese (DIO) rats. DIO rats were exposed to a high-fat diet with the supplementation of tart cherry seeds powder (DS) and seed powder plus juice (DJS). After 17 weeks, the DIO rats showed an increase of body weight, glycaemia, insulin, and systolic blood pressure. In the DS and DJS groups, there was a decrease of systolic blood pressure, glycaemia, triglycerides, and thiobarbituric reactive substances in the serum. In the DJS rats, computed tomography revealed a decrease in the spleen-to-liver attenuation ratio. Indeed, sections of the DIO rats presented hepatic injury characterized by steatosis, which was lower in the supplemented groups. In the liver of the DIO compared with rats fed with a standard diet (CHOW), a down-regulation of the GRP94 protein expression and a reduction of LC3- II/LC3-I ratio were found, indicating endoplasmic reticulum stress and impaired autophagy flux. Interestingly, tart cherry supplementation enhanced both unfolded protein response (UPR) and autophagy. This study suggests that tart cherry supplementation, although it did not reduce body weight in the DIO rats, prevented its related risk factors and liver steatosis.

## 1. Introduction

Obesity consists in the abnormal deposition of adipose tissue, associated with metabolic and chronic diseases, such as type-2 diabetes, heart diseases, hypertension, non-alcoholic fatty liver disease (NAFLD), and cancer [1]. Moreover, it is characterized by inflammation that alters cell metabolism and insulin signaling in metabolically active tissues [2].

The prevalence of obesity has reached epidemic proportions. Increased food intake, reduced physical activity, and altered metabolic processes affect energy balance, inducing obesity [3]. A high-fat diet (HFD) represents the etiology of obesity in modern societies. Therefore, the availability of useful animal models reflecting human obesity, such as diet-induced obese (DIO) rats, is crucial in the exploration of innovative compounds for the pharmacological treatment of obesity [4].

Many compounds present in fruit and vegetables have important nutraceutical properties, such as antioxidant and anti-inflammatory components. Indeed, natural bioactive compounds maintain low levels of reactive oxygen intermediates and inhibit the prostaglandin synthesis. Thus, they have been proposed as possible therapeutic tools for several diseases. For instance, anthocyanins are phytochemical flavonoids found in red-, blue-, and purple-pigmented fruits and vegetables. Anthocyanin is considered one of the flavonoids with a positive charge at the oxygen atom of the C-ring, which is a flavonoid basic structure [5]. Plenty of evidence suggests that anthocyanin-rich plant extracts modify lipid metabolism in vitro and reduce hyperlipidemia in vivo [6,7].

The current animal research suggests that tart cherry (*Prunus cerasus* L.) confers health benefits because it is an excellent source of anthocyanins. These compounds, cyanidin-3-glucosyl-utinoside, cyanidin-3-rutinoside, cyanidin-3-glucoside, and their a glycone, cyanidin, have exhibited in vitro antioxidant and cyclooxygenase inhibitory activities [8].

This study evaluates in DIO rats and the effects of the juice and seed powder of *Prunus cerasus* L., monitoring them for 17 weeks compared to age-matched control rats, fed with a standard diet (CHOW rats). Several techniques were performed to determine the possible protective effect of tart cherry supplementation against liver steatosis induced by obesity.

## 2. Materials and Methods

### 2.1. Animals and Diet

Male Wistar rats (Charles River; total *n* = 60; 250–275 g at the beginning of the experiments) of 7 weeks of age were used. The animals were housed individually, as previously described [9,10]. All procedures involving rats were conducted in accordance with the Institutional Guidelines, and were complied with the Italian Ministry of Health (protocol no. 1610/2013) and associated guidelines from European Communities Council Directive. The protocol was approved by the Ethics Committee of the University of Camerino (no. 7/2012, 6 June 2012). The CHOW rats (*n* = 24) were fed with standard laboratory diet *ad libitum* (4RF18, Mucedola, Settimo Milanese, Italy; 2.6 kcal/g), and the DIO rats (*n* = 36) were fed with a high-energy diet *ad libitum* (D12451, Research Diets, Inc., New Brunswick, NJ, USA; 4.73 kcal/g).

The CHOW and DIO rats were divided into the following three subgroups:

1. Control group (standard diet without supplementation);
2. CHOW and DIO rats supplemented with 0.1 mg/g/day of tart cherry seed powder (CS and DS, respectively);
3. CHOW and DIO rats supplemented with 0.1 mg/g/day of tart cherry seed powder plus tart cherry juice, containing 1 mg of anthocyanins (CJS and DJS, respectively).

Body weight and food intake were monitored every day. Systolic blood pressure was measured weekly.

Resistant rats (*n* = 6) were excluded from the study [9,11,12], because they did not develop an obese phenotype. 

After 17 weeks of supplementation, a computed tomography (CT) analysis was performed. Before the sacrifice, the systolic blood pressure was measured, and a blood sample was withdrawn from the tail vein. After the sacrifice, the liver was removed and washed in 0.1 M phosphate buffer saline (PBS). Portions of the liver were frozen at −80 °C for biochemical analysis. Other portions were placed in Bouin’s fluid for 6 h at room temperature and processed for paraffin embedding.

### 2.2. Preparation of Seeds Powder and Juice from Tart Cherries

The chemical, biological, functional, and technological properties of the sour cherry pomace and sour cherry seeds have already been described [13]. Previous studies have measured the total anthocyanins, total phenolic content, and Trolox equivalent antioxidant capacity in the sour cherry juice [14,15,16]. The sour cherry’s seed kernel contains vegetable oils including unsaturated fatty acids, oleic acids, α-tocopherol, tocotrienols, and tocopherol-like components. The components of its solid fraction include bioactive structures such as polyphenols, flavonoids, vegetable acids, and anthocyanidins. All of them have already been well characterized [17,18].

In our study, fresh tart cherries were pitted manually and mashed using a blender at room temperature for 5 min and then an Ultra Turrax for 1 min. The homogenate was then centrifuged at 7000× *g* for 10 min, and the extract was removed and stored at 4 °C until analyzed. The precipitate was further extracted in 96% ethanol for one night. The solution was centrifuged at 10,000× *g* for 20 min and the supernatant (ethanol extract) was collected and evaporated using a Rotary evaporator. The concentrated juice was added to the pulp extract and standardized, so that the rats could be given 1 mg of anthocyanins every day for 17 weeks [10]. The total monomeric anthocyanin content was measured using the differential method [19]. The juice was orally administered, using a standard water bottle. The dried seeds, deprived of the shell, were grounded and degreased with two ultrasound extraction rounds using 30 ml of petroleum ether. These seeds were incorporated into the standard diet giving to each animal 0.1 mg/g per day [17,18,19,20] for 17 weeks. For the DIO rats, 0.1 mg/g of seed powder was added to 1 g of lard.

### 2.3. Computed Tomography Analysis

The CT examinations of the abdomen were conducted with anesthetized rats positioned in dorsal recumbence, using a helical, single slice, multi detector scan (CT/e GE, Boston, MA, USA). The CT technical parameters were as follows: soft tissue acquisition algorithm, scan helical mode, slice thickness 1.0 mm, peak kilovoltage 120 kVp, X-ray tube current 100 mA, rotation time 1.5 s, starting from fourth cervical vertebra and proceeding caudally to pelvis.

CT images were acquired in a Digital Imaging and Communications in Medicine (DICOM) format and processed both with OsiriX (Pixmeo SARL, Berna, Switzerland) and ImageJ (http://rsb.info.nih.gov/ij/) software, as previously described [21]. Osirix software was used for the estimation of the subcutaneous fat layer, considering the thickness of both the subcutaneous and peritoneal fat layer at the level of the sternal xiphoid process, and for the assessment of the liver attenuation and calculation of the spleen-to-liver attenuation ratio, considering values >1 indicative of hepatic steatosis [22,23].

In addition, the same images were investigated with ImageJ software to assess the difference in hepatic optical density.

The DICOM images obtained with CT were converted into Tagged Image File Format (TIFF) images using Image J, and a 16-interval pseudo-color scale was applied to the grayscale. This scale starts from black pixels (value of 0), and increasing gradations of tissue density are represented in 16 equal intervals by a pseudo-color scheme to white pixels (value of 255). Within hepatic shadows, thirteen regions of interest (ROI; 3500 μm × 2400 μm), were evaluated. Hence, the distribution of pixels, in the same ROI, was calculated and displayed as a histogram.

### 2.4. Biochemical Analysis

First, 1000 µL of blood were collected in tubes with L-heparin. The blood samples were then centrifuged for 10 min at 3000 rpm. Glucose, total cholesterol, and triglycerides were evaluated by IDEXX Catalyst Dx. Insulin, and the thiobarbituric acid reactive substances (TBARS) concentration, expressed as the malondialdehyde (MDA) and superoxide dismutase (SOD) activity, were evaluated in the plasma with ^®^Ultrasensitive Rat Insulin ELISADRG (EIA-2943), TBARS assay Kit and SOD assay kit (both Cayman, Chemical Company, Ann Arbor, MI, USA), respectively, according to data sheets of the companies.

In the liver homogenates, the oxidation levels of the proteins were evaluated using an OxyBlot Protein Oxidation Detection Kit (Millipore, Merk, Darmstadt, Germany), according the manufacturer instructions.

### 2.5. Western Blot: Unfolded Protein Response (UPR) and Autophagy Analysis

The liver samples (0.1 ± 0.02 g) were lysed in lysis buffer containing protease inhibitor cocktail (Sigma Aldrich). Lysates were separated on 8%–14% Sodium Dodecyl Sulphate (SDS) polyacrylamide gel and transferred onto nitrocellulose membranes. The membranes were incubated overnight at 4 °C with the following primary antibodies: anti-caspase 3 (Cell Signaling Technology, Danvers, MA, USA, 1:1000), anti-Bcl-2-associated death (BAD), (Cell Signaling Technology, Danvers, MA, USA, 1:1000), anti-GRP94 (Cell Signaling Technology, 1:1000), anti-LC3 (Novus Biologicals, Centellian, CO, USA, 2 μg/mL), and anti- Glyceraldehyde 3-phosphate dehydrogenase GAPDH (Cell Signaling Technology, 1:1000; used as a loading control) [24].

### 2.6. Morphological Analysis

Hepatic tissue was removed from each rat. Consecutive sections (5 µm) of the liver, embedded in paraffin wax, were processed for morphological techniques and stained with haematoxylin and eosin (H&E). The sections were viewed under a light microscope. The images were transferred from the microscope by a DS-R12 NIKON camera and evaluated using a NIS-Elements Nikon image analyzer. Blinded researchers to the group distributions performed the histological analyses of the slides using light microscopy at 20× magnification, evaluating in different fields of 300,000 μm^2^ 400 hepatocytes in alternative slides. For each field to validate the histological features and to determine the hepatic steatosis, a scoring system was applied [25]. Briefly, the steatosis scores were defined as follows: the presence of intrahepatic fat droplets in <5% of hepatocytes for field (score 0), the presence of intrahepatic fat droplets in 5%–33% of hepatocytes (score 1), the presence of intrahepatic fat droplets in 33%–66% of hepatocytes (score 2), and the presence of intrahepatic fat droplets in >67% of hepatocytes as score 3.

### 2.7. Statistical Analysis

All of the results were expressed as mean ± standard error of the mean (SEM). Regarding the food intake and body weight, data were analyzed using two-way analysis of variance (ANOVA) with the animal group as the between-subject variable and time as the within-subject variable, followed by post hoc comparison carried out by the Bonferroni test. For the others parameters, the data were analyzed by two-way ANOVA, with the animal group as the between-subject variable, followed by a post hoc comparison carried out by the Bonferroni test. The *p*-values < 0.05 were considered statistically significant.

## 3. Results

### 3.1. Body Weight, Food Intake, and Systolic Blood Pressure

At the beginning, the body weight of the rats assigned to the high fat diet (HFD), DIO group (298.1 ± 2.7 g), did not differ significantly from that of the rats in the control CHOW group (297.7 ± 2.1 g; *p* > 0.05 vs. DIO rats).

**Figure 1:**
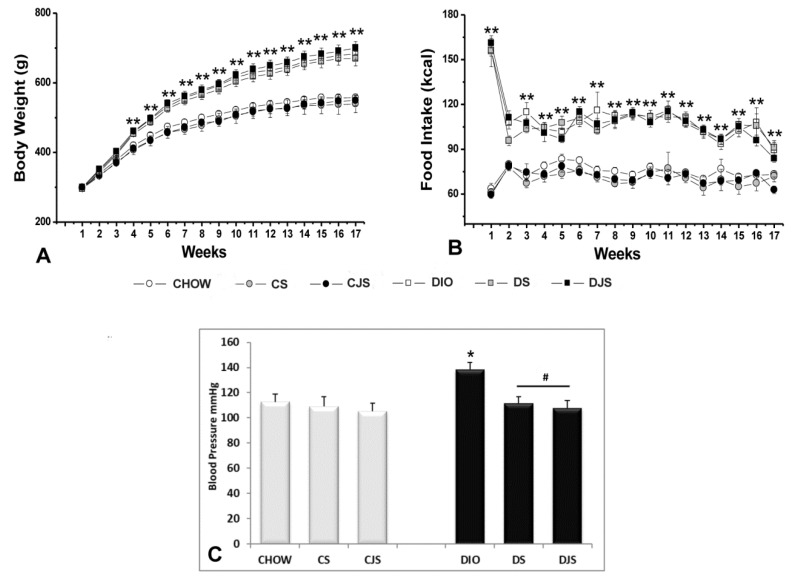
(**A**) Body weight in grams (g). (**B**) Food intake in kilocalories (kcal), measured weekly in all of the animal groups. ** *p* < 0.01 *vs*. CHOW. (**C**) Systolic blood pressure in mmHg measured at the end of the study in all of the animal groups. CHOW—control rats with standard diet; CS—CHOW plus tart cherry seeds; CJS—CHOW plus tart cherry seeds and juice; DIO—diet-induced obese rats; DS—DIO plus tart cherry seeds; DJS—DIO plus tart cherry seeds and juice. Data are the mean ± standard error of the mean (SEM). * *p* < 0.05 vs. CHOW; # *p* < 0.05 vs. DIO.

After 17 weeks, the overall ANOVA showed a significant difference in body weight between the CHOW and DIO groups (*p* < 0.01); the post-hoc test showed that body weight of DIO rats was significantly higher in comparison with the CHOW rats, starting from the fourth week (*p* < 0.01). Significant differences among the groups evaluated by the post hoc analyses are indicated in Figure 1A. The overall ANOVA showed a significant difference in energy intake (kcal) between the groups (*p* < 0.01). Post-hoc differences are shown in Figure 1B. In the CS, CJS, DS, and DJS groups the supplementation did not affect body weight or food intake (Figure 1A,B).

Systolic blood pressure was higher in the DIO rats after 17 weeks of HFD compared with the age-matched CHOW rats. The DS and DJS rats showed a significant reduction of systolic blood pressure compared with DIO rats (Figure 1C).

### 3.2. Blood Parameters

**Figure 2:**
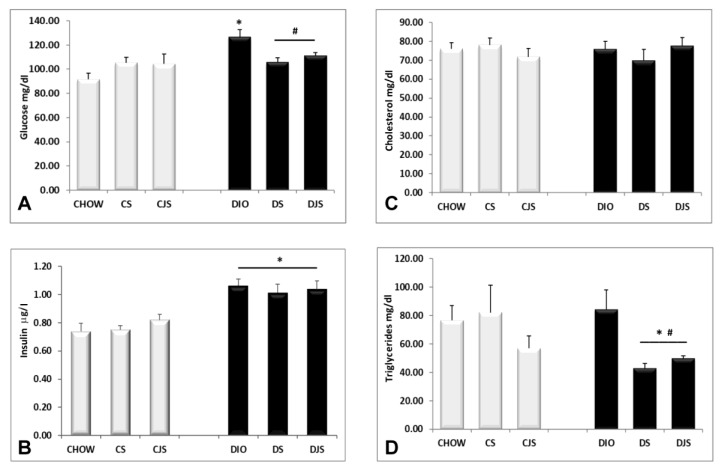
Blood parameters. Levels of (**A**) glucose in mg/dL, (**B**) insulin in μg/L, (**C**) cholesterol, and (**D**) triglycerides in mg/dL, measured at the end of the study in all of the animal groups. CHOW—control rats with standard diet; CS—CHOW plus tart cherry seeds; CJS—CHOW plus tart cherry seeds and juice; DIO—diet-induced obese rats; DS—DIO plus tart cherry seeds; DJS—DIO plus tart cherry seeds and juice. Data are the mean ± SEM. * *p* < 0.05 vs. CHOW rats; # *p* < 0.05 vs. DIO rats.

The obese condition induced an increase of glucose (*p* < 0.05, Figure 2A) and insulin (*p* < 0.05, Figure 2B) levels in the DIO rats after 17 weeks of HFD. Tart cherry supplementation reduced only hyperglycemia (Figure 2A), but not the hyperinsulinemia (Figure 2B). Obesity did not significantly affect the total cholesterol (Figure 2C) and triglycerides (Figure 2D) levels. However, the tart cherry intake significantly reduced the blood level of the triglycerides in the treated DIO rats compared with the DIO control rats (*p* < 0.05, Figure 2D).

**Figure 3:**
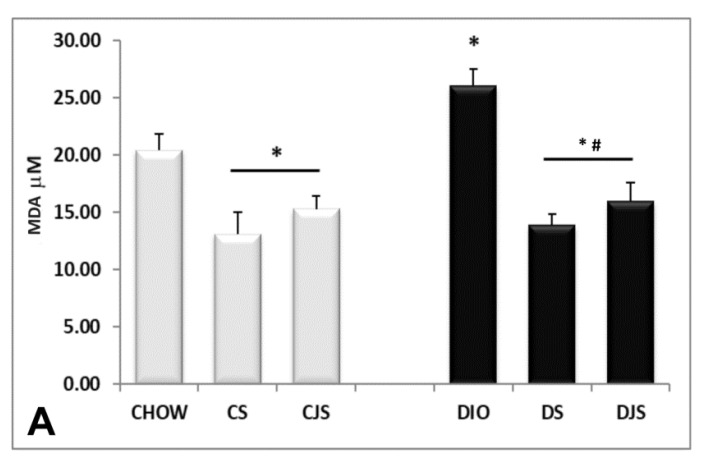

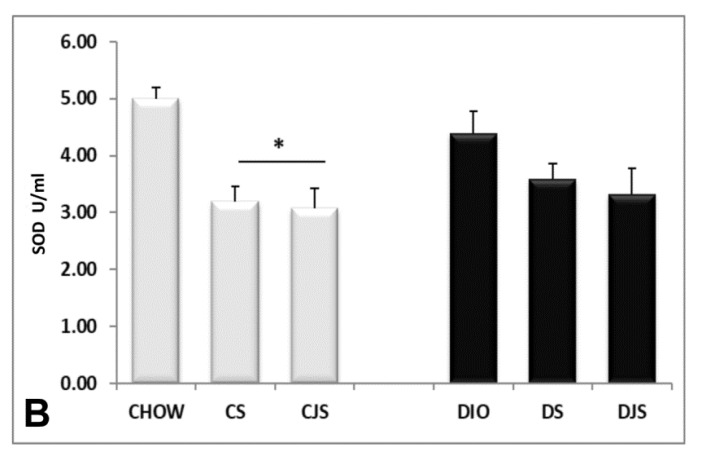
Concentrations of (**A**) malondialdehyde (MDA) and (**B**) superoxide dismutase (SOD) activity in the serum. MDA concentrations are expressed in μM and SOD activity are expressed in unit/mL(U/mL), where one unit is defined as amount of enzyme needed to exhibit 50% dismutation of the superoxide radicals. CHOW—control rats with standard diet; CS—CHOW plus tart cherry seeds; CJS—CHOW plus tart cherry seeds and juice; DIO—diet-induced obese rats; DS—DIO plus tart cherry seeds; DJS—DIO plus tart cherry seeds and juice. Data are the mean ± SEM. * *p* < 0.05 vs. CHOW rats; # *p* < 0.05 vs. DIO rats.

The TBARS assay kit revealed an increase of MDA in the serum of the DIO rats (Figure 3A) compared with the control CHOW group (*p* < 0.05), indicating a condition of lipid peroxidation induced by oxidative stress [26]. The levels of MDA decreased in the serum of the DS and DJS groups (*p* < 0.05, Figure 3A), suggesting a reduction of oxidative stress with the supplementation of tart cherries [27]. SOD activity was found in the CHOW and DIO rats, decreasing significantly only in the CS and CJS rats compared with the CHOW group (*p* < 0.05, Figure 3B).

### 3.3. CT Evaluation

In the CT, fat infarction of the liver was investigated. For the assessment, the difference in attenuation values between the liver and the spleen, as well as the calculation of the spleen-to-liver attenuation ratio were taken into account. At 17 weeks, in the DIO rats, liver attenuation was 10 HU (Hounsfield units) smaller than the spleen, while in the CHOW rats, the attenuation values of the liver (65 HU) and spleen (61 HU) were the same.

**Figure 4:**
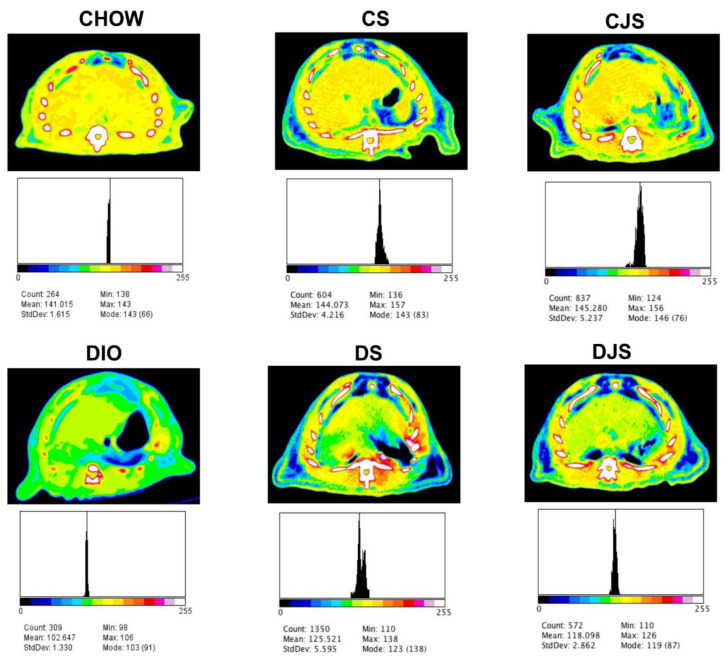
Computed tomography (CT) images evaluation with Image J software. The grayscale Digital Imaging and Communications in Medicine (DICOM) images obtained with CT were converted into pseudo-color images, which starts with the lowest density of black pixels (value of 0, air) and increases gradually, depending on the tissue density, to white pixels (value of 255, bone). The hepatic optical density for the different groups of rats ranged from 102.647 to 145.280, as reported below each image. The black peak in each histogram indicates the distribution of the optical density of the regions of interest investigated. CHOW—control rats with standard diet; CS—CHOW plus tart cherry seeds; CJS—CHOW plus tart cherry seeds and juice; DIO—diet-induced obese rats; DS—DIO plus tart cherry seeds; DJS—DIO plus tart cherry seeds and juice.

Regarding the spleen-to-liver attenuation ratio, it was 1.06 ± 0.03 in CHOW rats, while in the DIO and DS rats it was significantly higher: 1.18 ± 0.04 and 1.18 ± 0.08, respectively (*p* < 0.05 vs. CHOW rats). This ratio decreased remarkably only in the DJS group rats (0.95 ± 0.07; *p* < 0.05 vs. DIO rats). An analysis of the hepatic optical density (Figure 4) revealed a similar liver density for CHOW, CS, and CJS rats, with mean pseudo-color density values of 141.01 ± 1.61, 144.07 ± 4.21, and 145.28 ± 5.23, respectively. On the other hand, the DIO rats showed the lowest hepatic density (102.64 ± 1.33), while the DS and DJS rats had a similar hepatic optical density, with values of 125.52 ± 5.59 and 118.09 ± 2.86, respectively.

### 3.4. Liver Morphology

**Figure 5:**
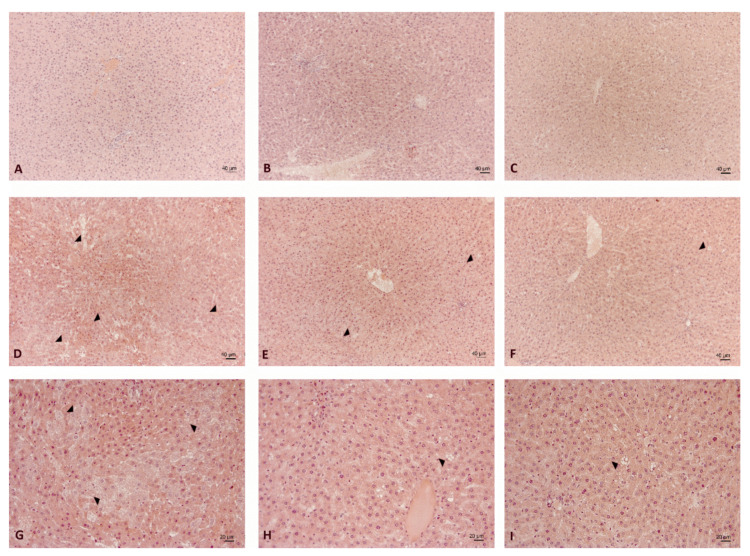
Liver morphology. Representative sections of liver processed for haematoxylin and eosin staining of (**A**) CHOW, (**B**) CS, (**C**) CJS, (**D**,**G**) DIO, (**E**,**H**) DS, and (**F**,**I**) DJS rats. (**A**–**F**). Magnification 10×. Calibration bar: 40 μm. (**G**–**I**) Magnification 20×. Calibration bar: 20 μm. Arrow heads indicate features of steatosis, differently occurring in the experimental groups examined. CHOW—control rats with standard diet; CS—CHOW plus tart cherry seeds; CJS—CHOW plus tart cherry seeds and juice; DIO—diet-induced obese rats; DS—DIO plus tart cherry seeds; DJS—DIO plus tart cherry seeds and juice.

In the CHOW rats, the liver morphology was normal and well preserved, independent of tart cherry supplementation (Figure 5A–C). Rarely, the intake of powder seed alone or powder seed plus sour juice induced alterations in the hepatic lobule characterized by dilated sinusoids. The regularity of these changes appeared greater in the CJS rats (Figure 5C).

In the DIO rats, the morphological pattern showed alterations in the hepatic structure with different degrees of severity; it was scored as 2.1 ± 0.2 for steatosis, compared with the CHOW liver, which scored as 0 (Figure 5D,G). Dilated sinusoids and hepatocytes with no homogeneous staining were found in some areas: strongly eosinophilic cells were observed, between weakly staining hepatocytes.

**Figure 6:**
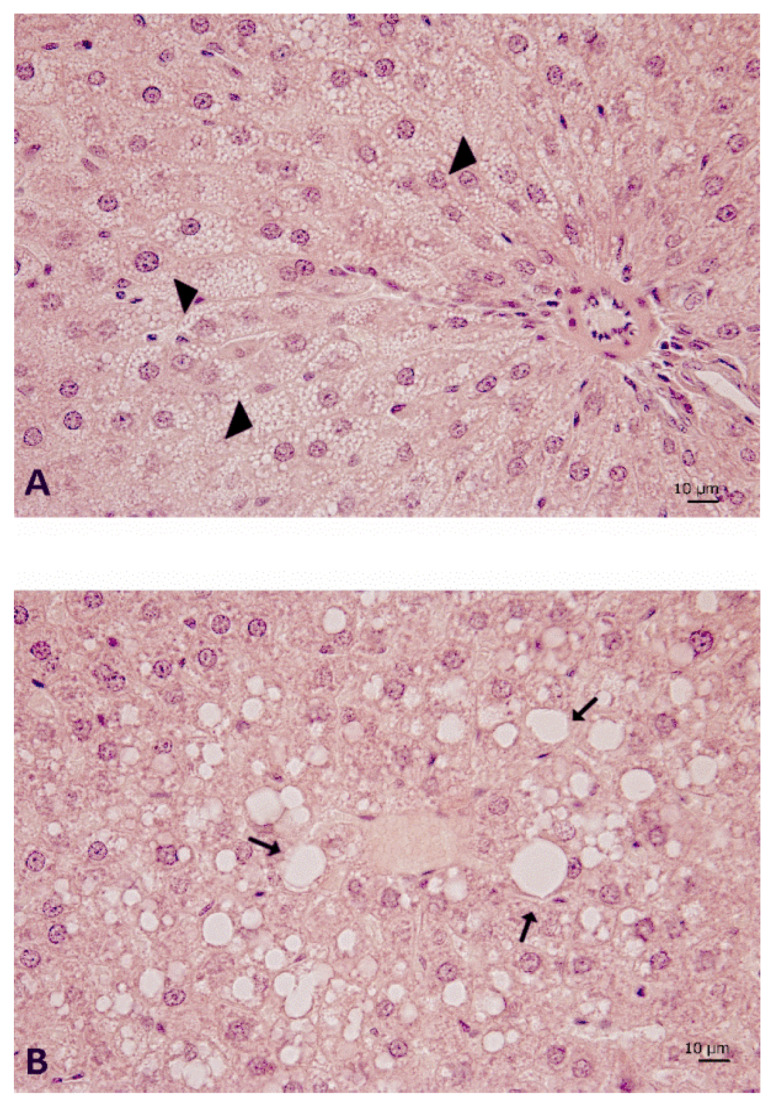
Liver morphology of DIO rats. Sections of liver processed for haematoxylin and eosin staining to highlight the (**A**) microvesicular steatosis, mainly found in the periportal areas (arrow head), and (**B**) macrovesicular steatosis (arrow), in a centrilobular area where several scattered balloon cells can be often observed. Magnification 40×. Calibration bar: 10 μm. DIO—diet-induced obese rats.

The typical features of steatosis are identifiable in some lobular areas both in the microvesicular (Figure 6A) and macrovesicular elements (Figure 6B). The presence of large vacuolizations was especially evident in the centrolobular areas (Figure 6B). From a morphological point of view, this structural alteration appeared less evident in the DS (Figure 5E,H) and DJS rats (Figure 5F,I) scored as 0.8 ± 0.1 and 0.4 ± 0.2 for steatosis, respectively. Only a few elements of steatosis were present in the hepatocytes around the central veins in the DS (Figure 5H) and DJS rats (Figure 5I).

### 3.5. Oxidative Stress Condition in the Liver

**Figure 7:**
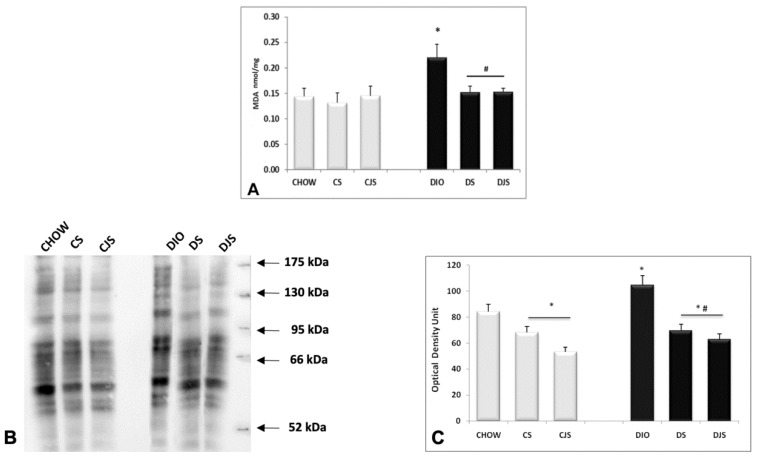
Concentrations of (**A**) malondialdehyde (MDA) in the liver expressed in nmol/mg of tissue. (**B**) OxyBlot in the liver homogenates and (**C**) the graph reports the values of optical density measured in the arbitrary optical density unit. CHOW—control rats with standard diet; CS—CHOW plus tart cherry seeds; CJS—CHOW plus tart cherry seeds and juice; DIO—diet-induced obese rats; DS—DIO plus tart cherry seeds; DJS—DIO plus tart cherry seeds and juice. Data are the mean ± SEM. * *p* < 0.05 vs. CHOW rats; # *p* < 0.05 vs. DIO rats.

The TBARS kit analysis revealed in the liver homogenates an increase of MDA in DIO rats (*p* < 0.05), compared with the CHOW group, demonstrating a condition of oxidative stress in obesity (Figure 7A). The levels of MDA decreased in the DS and DJS rats (*p* < 0.05, Figure 7A), indicating a reduction of oxidative stress in the presence of tart cherries in the liver. Moreover, an increased density of oxidized proteins at different molecular weight was detected in the DIO rats compared with the CHOW rats (Figure 7B,C). Tart cherry juice and seeds decreased the level of oxidized proteins, both in lean and obese rats (Figure 7B,C).

### 3.6. ER Stress and Autophagy Evaluation in Obesity Condition

**Figure 8:**
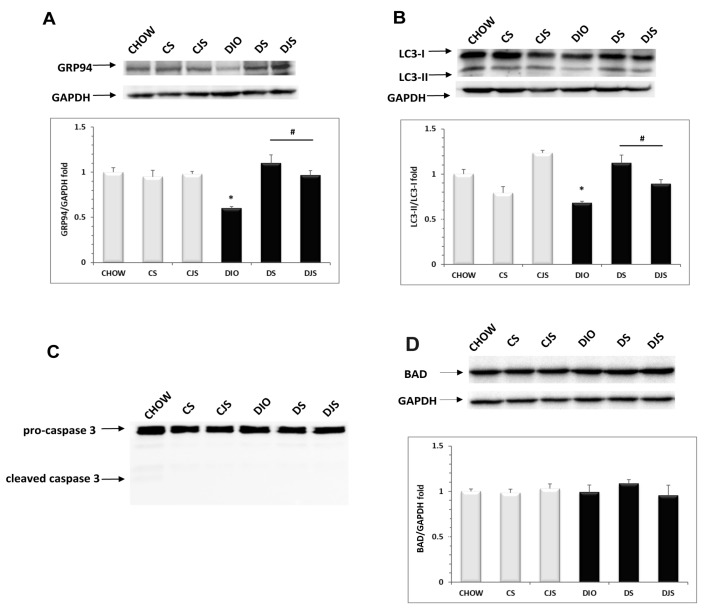
Lysates of liver from all of the animal groups were immunoblotted using specific antibodies, namely: (**A**) anti-GRP94 molecular weight 100 KDa; (**B**) anti-LC3 that recognized the isoform at 16 KDa (LC3-I) and 14 KDa (LC3-II); (**C**) anti-caspase 3 that revealed the pro-caspase 3 at 31 KDa and cleaved caspase 3 at 17 KDa; and (**D**) anti-BAD that revealed a band at 20 KDa. Values indicate the densitometric analysis using CHOW rats as the control. GAPDH levels were used as the loading control. Blots are representative of three separate experiments. The LC3-II/LC3-I ratios were calculated after densitometric analysis. CHOW—control rats with standard diet; CS—CHOW plus tart cherry seeds; CJS—CHOW plus tart cherry seeds and juice; DIO—diet-induced obese rats; DS—DIO plus tart cherry seeds; DJS—DIO plus tart cherry seeds and juice. * *p* < 0.05 vs. CHOW rats; # *p* < 0.05 vs. DIO rats.

Using Western blot analysis, we demonstrated a marked down-regulation of GRP94 protein expression (evaluated with a band of approximately 100KDa) in DIO rats compared with CHOW rats (*p* < 0.05, Figure 8A), suggesting that obesity induces an unfolded protein response (UPR) impairment. In the DS and DJS groups, tart cherry supplementation restored the normal GRP94 protein levels, improving the efficiency of the chaperone protein in the endoplasmic reticulum (ER). No changes in the GRP94 expression levels were found in the CS or CJS rats compared with control animals (Figure 8A). Moreover, we assessed the autophagic process by evaluating the LC3 turnover (Figure 8B). Indeed, during autophagy, the cytosolic form of LC3 (LC3-I, molecular weight 16 KDa) was conjugated with phosphatidylethanolamine to form a LC3–phosphatidylethanolamine conjugate (LC3-II, molecular weight 14 KDa), which is recruited to autophagosomal membranes. Thus, the LC3-II/LC3-I ratio was used to investigate autophagy [28]. We showed that in DIO rats, the LC3-II/LC3-I ratio was reduced with respect to the control (Figure 8B), indicating an impairment in the autophagic flux, completely reverted by both tart cherry supplementations.

UPR and autophagy dysfunctions are associated with apoptotic cell death [29]. Therefore, we investigated the apoptosis induction by caspase 3 activation assessment and BAD protein expression. Our results showed that no cleavage of caspase 3 was present in any of the samples (Figure 8C) and no change in the expression of the BAD protein was observed (Figure 8D), suggesting that liver injury induced by diet is associated with hepatocyte cell damage, but not with cell death.

## 4. Discussion

Obesity is a multifactorial disease that has reached an epidemic level all over the world. Overweight, besides increasing the risk for several diseases, promotes the inflammatory status leading to negative consequences, such as NAFLD or hypertriglyceridemia due to insulin resistance [30,31,32].

Diet-induced obesity in rats provides a suitable animal model, sharing several common features with human obesity [33]. Our findings confirmed that four weeks of exposure to HFD led to a significant increase in body weight in DIO rats compared with control rats. The supplementation of *Prunus cerasus* L. did not reduce this weight gain, demonstrating that tart cherry did not prevent fat accumulation induced by HFD (45%) *ad libitum*. This result is in agreement with other studies, in which oral anthocyanin treatment did not preserve the rats from diet induced weight gain [34,35,36], and they did not affect the hypertrophy of adipocytes during the development of obesity [37].

The effects of anthocyanins supplementation on body weight were controversial. HFD supplemented with purified anthocyanins from blueberries reduced weight gain and fat accumulation in C57BL/6 mice [38]. In contrast, lyophilized wild blueberry powder intake induced body fat accumulation [38]. Moreover, it has been reported that the ingestion of blueberry juice did not significantly reduce the body weight gain and the weight of white adipose tissue in mice fed with HFD [39].

In the present study, the values of glycemia and insulin were higher in the DIO rats compared with the control CHOW rats, indicating a condition of insulin resistance typical of type-2 diabetes mellitus. However, the DIO rats did not show hyperlipidemia. Indeed, the levels of triglycerides and total cholesterol were similar to the CHOW rats, and they did not increase after 17 weeks under HFD. Rats are generally more resistant to developing hypercholesterolemia and the relative atherosclerosis [40,41,42]. In fact, to establish a condition of hyperlipidemia, it is necessary for the use of a specific high cholesterol diet [43,44] or a longer period of HFD consumption [41,42].

Regarding supplementation, tart cherries decreased triglycerides in DIO rats, but not in CHOW. In accordance with other studies, tart cherry did not modify the lipids level, including triglycerides, in a healthy condition [45,46], on the contrary it did in an overweight and obesity condition in human and animal models [47,48,49].

A transient increase in fatty acid levels can also be considered as the physiological stimulation of insulin production as insulin secretion that may temporarily increase to maintain a metabolic balance. However, a prolonged fatty acid overload impairs β cell function [36]. In line with our study, the hypercaloric diet showed increasing insulin secretion, while the anthocyanin treatment failed to counteract the HFD effect on insulin secretion [36]. Among the anthocyanins, cyanidin-3-o-β glycoside has been revealed to be able to increase the cellular insulin sensitivity through the inactivation of Jun NH(2)-terminal kinase (JNK) or not converting the serine insulin receptor substrate-1 [50]. Moreover, DIO rats developed a condition of hypertension, compared with the CHOW rats. High blood pressure represents one of the main risk factors for liver injury and hepatic fibrosis [51]. Although tart cherry supplementation did not reduce the increase of body weight in DIO rats, it reduced the oxidative stress condition, the systolic blood pressure, and glycemia values, confirming the positive effects of tart cherry intake on the risk factors of obesity and metabolic syndrome [27,49,52].

The liver plays a central role in the metabolism, especially in the lipid one. Because of the delivered and stored lipoprotein imbalances, stress, or injuries, there could be some form of lipid depots inside the hepatocytes of the liver parenchyma [53,54]. An infiltration of fat higher than 5%, without a history of alcohol abuse, has often used as the definition of NAFLD [55]. The prevalence of this pathology is also influenced by age, gender, ethnicity, sleep apnoea, and endocrine dysfunction [56,57,58].

In the development of hepatic steatosis, the metabolic syndrome, especially insulin resistance, has a major influence on obesity alone, even if hepatic steatosis is strongly associated with visceral adiposity. This because hyperinsulinemia promotes both lipogenesis, hepatocytes fat accumulation, and lipolysis, with hyperlipidemia [55,59,60].

Studies evaluating the effect of anthocyanins in vivo on hepatic lipid metabolism, steatosis, oxidative stress, and steatohepatitis have been previously reviewed, also with a certain difficulty because of the very different experimental models and the dissimilar outcomes for the assessment of lipid metabolism, oxidative stress, and liver injury [61]. Anyway, the intake of tart cherries rich in anthocyanins prevented the development of metabolic alterations in insulin-resistant rats [49] or in a high-fat/high-fructose (HFHF) model of diabetic-rats [62]. Hence, it was described that the cherry-enriched diet reduced the fatty liver, and that anthocyanin-rich extracts may exert positive effects enhancing the activity of the hepatic peroxisome proliferator-activated receptor alpha (PPAR-α) and PPAR-α target acyl-coenzyme A oxidase mRNA [49]. Specifically, cyanidin was found to act as an agonistic ligand for PPAR-α, and it reduced the hepatic lipids, regulating the genes involved in lipid metabolic pathways [63]. It was postulated that PPARα promoted the lipolysis and reduced lipogenesis, and as a consequence, the hepatic fat content decreased [61]. Moreover, cherry consumption reduced steatosis, as described in type 2 diabetes rats, through the inhibition of the activation of the sterol regulatory element-binding proteins and carbohydrate-responsive element-binding proteins [62].

In our study, the steatotic alterations related to obesity and the positive effects of tart cherry intake were also demonstrated by CT analysis. In human beings, values of the liver 10 HU smaller than those of the spleen are considered highly predictive of hepatic steatosis. Spleen-to-liver attenuation ratios greater than 1.1 are proposed to be suggestive for hepatic steatosis, even if liver biopsy is still considered as the gold standard in the assessment of fatty infiltration [64]. In the present study, spleen to liver attenuation was not able to suggest hepatic steatosis in DS rats, while the hepatic optical density gave similar results in both DS and DJS rats, confirmed by the liver morphology analysis. The CT evaluation of the attenuation difference between liver to spleen represents an important technique to investigate the association between fatty liver and visceral fat in humans [23,55]. Similarly, inrats, CT scans diagnose and quantify the degree of liver fat infiltration [22].

To the best of our knowledge, this was the first attempt to apply non-invasive diagnostic indexes such as spleen-to-liver attenuation and hepatic optical density in rats, and further studies are needed to identify the specific cut-off points for steatosis in rats.

The CT results were directly correlated with the histological evidence. Indeed, both the DS and DJS groups showed a reduction in the steatosis, persisting only in limited portions of tissue and in the microvesicular form. These positive effects could be due to the reduction of oxidative stress. It was demonstrated that in the liver, cherry consumption decreased oxidative stress, through the inhibition of the NADPH oxidase subunit p22phox expression, nuclear factor erythroid-2 related factor 2 (Nrf2) degradation, and the formation of reactive oxygen species [62]. Studies reported that anthocyanins (i.e., cyanidin-3-O-β-glucoside) may avoid the development of liver impairment, reducing the lipid peroxidation [65] or the oxidative stress by the induction of anti-oxidant enzymes [66]. However, whether an enhanced redox status was secondary or independent of the reduced hepatic lipids and an improved metabolic status was not established [61]. Both the seeds and juice reduced inflammation and oxidative stress in cell lines and weight in obese subjects in a randomized, crossover pilot study [67,68,69].

Several findings demonstrated that ER stress develops in the liver of obese animals, where it plays an important role in hepatic lipogenesis [70]. During ER stress, the UPR pathways are activated to remove the polypeptides that fail to reach the appropriate folding, in order to restore the ER homeostasis. It has been recently demonstrated that UPR dysfunction prolongs the ER stress in the liver and induces the development of hepatic steatosis [71]. In agreement with these results, we demonstrated a marked down-regulation of the expression levels of GRP94, a chaperone belonging to the Hsp90 family, in DIO rats, indicating an impairment of the UPR pathway. Interestingly, tart cherry supplementations restored the GRP94 protein levels, showing the ability to improve the recovery of the ER function. Moreover, it is well known that autophagy, a pathway responsible for the degradation of unwanted or damaged cytoplasmic organelles, is involved in the control of hepatic lipid droplets under stress conditions such as obesity. It is responsible for the degradation of lipid droplets. In fact, its inhibition was found to increase the triglycerides contents in hepatocytes [72]. In line with these results, our data show that the obesity promoted autophagy impairment. Tart cherry supplementations markedly improved the autophagic flux, as evidenced by the LC3-II/LC3-I ratio. As ER stress or autophagy inhibition lead to apoptosis, we also evaluated this process. Similar to previous findings in diabetic mice with liver injuries [71], no apoptosis was observed in the liver of the DIO animals, suggesting that obesity induced liver damage promotes lipid accumulation in hepatocytes but not hepatocyte apoptotic cell death.

Thus, overall, our results demonstrated that the dysfunctions of UPR and autophagy lead to the impairment of lipid droplet movement in hepatocytes, contributing to the exacerbation of the steatohepatitis, and that tart cherry supplementations represent a good strategy to restore hepatocyte cell homeostasis during obesity.

## 5. Conclusions

The present study revealed that the *Prunus cerasus* L. seeds and juice could decrease oxidative stress and steatosis in the liver of DIO rats. Further studies are needed to clarify their potential utility as bioactive compounds of the anthocyanins, flavonoid compounds, vegetable oils including unsaturated fatty acids, and oleic acids composing juice and seeds, respectively.
